# Investigation of *PAG2* mRNA Expression in Water Buffalo Peripheral Blood Mononuclear Cells and Polymorphonuclear Leukocytes from Maternal Blood at the Peri-Implantation Period

**DOI:** 10.3390/vetsci6010008

**Published:** 2019-01-14

**Authors:** Olimpia Barbato, Gabriella Guelfi, Laura Menchetti, Gabriele Brecchia, Noelita Melo de Sousa, Claudio Canali, Francesco Grandoni, Maria Carmela Scatà, Giovanna De Matteis, Anna Beatrice Casano, Jean François Beckers, Vittoria Lucia Barile

**Affiliations:** 1 Dipartimento di Medicina Veterinaria, Università degli studi di Perugia, 06100 Perugia, Italy; gabriella.guelfi@unipg.it (G.G.); laura.menchetti7@gmail.com (L.M.); gabriele.brecchia@unipg.it (G.B.); claudio.canali@unipg.it (C.C.); annabeatrice.casano@libero.it (A.B.C.); 2 Laboratoires d’Endocrinologie Animale et de Reproduction, Faculté de Médicine Vétérinaire, Université de Liège, B-4000 Liège, Belgium; noelitamelo@gmail.com (N.M.d.S.); jfbeckers@ulg.ac.be (J.F.B.); 3 CREA Consiglio per la ricerca in agricoltura e l’analisi dell’economia agraria, Centro di ricerca Zootecnia e Acquacoltura, 00015 Monterotondo (Roma), Italy; francesco.grandoni@crea.gov.it (F.G.); mariacarmela.scata@crea.gov.it (M.C.S.); giovanna.dematteis@crea.gov.it (G.D.M.); vittorialucia.barile@crea.gov.it (V.L.B.)

**Keywords:** *PAG2* mRNA, peri-implantation, pregnancy diagnosis, water buffalo

## Abstract

The main objective of this study was to assess *PAG2* mRNA expression in maternal blood cells at the peri-implantation period in water buffalo; moreover, we wanted to evaluate the earliest time in which PAG-2 could be detected in maternal blood. Thirty-two lactating buffaloes artificially inseminated (AI) were utilized. Blood was collected at Days 0, 14, 18, 28, 40 after AI (AI = day 0). Pregnancy was diagnosed by ultrasound at Days 28 and 40 post AI. Out of 32 buffaloes, 14 were pregnant (P group) and 18 were not pregnant (NP group). The plasma PAG-2 threshold of 1.0 ng/mL in the P group was reached at day 40 post AI. *PAG2* mRNA expression differed between the P and NP groups, and was either evaluated in Peripheral Blood Mononuclear Cells (PBMC) or Polymorphonuclear Leukocytes (PMN), starting from day 14. However, both the estimated marginal means and multiple comparisons showed that *PAG2* mRNA expression was higher in PMN than PBMC. In the present study, PAG-2 appeared in the blood (40 Days post AI), and an early expression of *PAG2* mRNA at Day 14 post AI was also observed. Although further research is undoubtedly required, *PAG2* mRNA in peripheral blood leukocytes could be using to better understand the role that PAGs play during pregnancy in buffalo.

## 1. Introduction

Pregnancy-associated glycoproteins (PAGs) constitute a large aspartic Proteinase family of glycoproteins expressed in the outer epithelial layer of the placenta in eutherian species [1,2,3], and are particularly numerous in ruminant ungulates. They are probably involved in proper placenta development and embryo maternal interaction in Eutherian mammals.

In ruminants, the PAG gene family is particularly large and complex. Dozens of distinct cDNAs, and numerous variants, have been cloned from cattle, sheep, goat, bison, buffalo and deer placentae [4,5,6,7,8,9,10,11,12,13,14,15,16]. The PAG gene family in ruminants is comprised of two evolutionarily distinct groups [8,17,18]. Based on the time when each group arose, phylogenetically PAGs can be defined as ancient (PAG-2), that is, they originated 87 million years ago, and modern (PAG-1) that are estimated to have originated 52 million years ago [8,13,17,19]. Bovine PAG-2 coexists with bovine pregnancy-associated glycoprotein 1 in the trophectoderm [8,19,20,21] and their functions are not completely understood [20,22]. PAG-1 are produced in binucleate cells of both intercotiledonary and cotyledonary chorion, while PAG-2 molecules are produced by both mononucleate and binucleate trophoblastic cells [23]. The majority of PAGs belong to the modern group and these have only been observed in the Ruminantia [13,21]. Although the functions of these glycoproteins are not yet fully understood, plasma PAG-I concentrations have been used for pregnancy diagnosis and as a marker of placental/fetal well-being [6,21,24,25,26,27]. Water buffalo PAG-1 concentrations can be detected in the blood of pregnant females around Day 28 and 30 of gestation by radioimmunoassay (RIA) [15,28,29,30]. Very recently, a new approach was described in which an antiserum was raised against boPAG-2 allowing PAG molecules to be detected in maternal blood [31,32,33,34,35] in bovine species. For the buffalo species, eleven PAG molecules from group-I were purified and characterized from water buffalo placenta (wbPAG) [15,28,29,30] while 19 different complementary DNA (cDNA) of PAG genes were identified in this species [36]. With regard to expression of the PAG-II group, by using real time PCR technique, it was demonstrated that *PAG2* mRNA can be detected in placenta as early as 17–19 days of pregnancy, coinciding with the beginning of implantation in the bovine placenta [3], 18 days in caprine [7], and 13 days in ovine [8]. Barbato et al. [36] were the first to investigate mRNA PAG-2 expression in blood during early pregnancy in water buffalo. No investigation have been carried out on the quantification of *PAG2* mRNA expression in specific blood cells.

The main objective of this study was to assess *PAG2* mRNA expression in the maternal subset of blood leukocytes at the peri-implantation period in water buffalo; moreover, we wanted to evaluate the earliest time in which PAG-2 could be detected in maternal blood.

## 2. Materials and Methods

### 2.1. Animals and Experimental Design

This study was carried out at the CREA Centro di ricerca Zootecnia e Acquacoltura experimental farm, located near Rome, Italy (42°3′ N, 12°37′ E), from February to April 2015 during the daylight-lengthening period, which represents the transition from the breeding to the nonbreeding season for this species. The animals involved in this trial were supervised in compliance with Italian laws and regulations regarding experimental animals. The experimental design was performed according to good veterinary practices under farm conditions. The CREA Centro di ricerca Zootecnia e Acquacoltura is authorized to use farm animals for experimental design (as stated in DM 26/96-4 of Italian Welfare Ministry). Thirty-two lactating Italian Mediterranean buffalo cows were used to determinate the PAG concentrations and RNA isolation. The animals were housed in an open paddock, fed ad libitum on total mixed rations based on maize silage, alfalfa hay, soya bean meal, maize meal and barley meal (containing 0.90 UFL/Kg of dry matter (DM) and 15% crude protein on DM) and milked twice daily.

The buffalo cows were treated with a progesterone-releasing intravaginal device (PRID^®^; Sanofi, France), containing 1.55 g natural progesterone kept in place for 10 days. At the time of PRID withdrawal an i.m. injection of 500 IU of PMSG (Ciclogonina^®^, Pfizer, Latina, Italy) and 0.15 mg of cloprostenol (PGF_2α_ analogue) (Dalmazin^®^, FATRO, Ozzano Emilia, Bologna, Italy) were given. The evening before artificial insemination (AI) buffaloes were treated intramuscularly with 150-ug gonadorelin (GnRH, Enagon; Intervet, Aprilia, Latina, Italy). Animals were artificially inseminated using frozen-thawed semen at 72 h after PRID^®^ removal. 

Blood was collected from the jugular vein into 10 mL EDTA coated tubes at Days 0, 14, 18, 28 and 40 after AI (AI = day 0). Day 0 was considered as the control day. For cell isolation, blood samples were immediately processed. The isolated Peripheral Blood Mononuclear Cell (PBMC) and Polymorphonuclear Leukocytes (PMN) were stored at −80 °C until qRNA extraction. For PAG-2 determination, plasma was separated by centrifugation at 2500× g for 10 min, and stored at −20 °C until assayed. 

### 2.2. Pregnancy Diagnosis

Pregnancy diagnosis was performed on Day 28 and Day 40 after AI by transrectal ultrasonography (Aloka–SSD Prosound 2 scanner, Hitachi Medical System, Italy) with a 7.5 MHz linear-array transducer. All ultrasound scans were performed by the same operator. Positive pregnancy status at Day 28 and Day 40 were characterized by the presence of embryonic vesicles and embryo proper within the vesicle and the heartbeat visualization (embryo viability) [37]. In the absence of embryonic vesicle or embryo proper in the uterine lumen at Day 28 and Day 40, females were considered as non-pregnant. 

### 2.3. PAG-2 Radioimmunoassay

The bovine PAG-2 radioimmunoassay used has been described recently in [31,34,38]. Briefly, PAG-2 was purified according to the method detailed by Beckers et al. [39]. The primary antibody was rabbit polyclonal antiserum against boPAG-2 (AS#438) raised as described by Vaitukaitis et al. [40]. Owing to the instability of the boPAG-2 molecule, boPAG-1 (67 kDa) was used as a standard (dilutions ranging from 100 to 0.2 ng/mL) and for iodination with the 125-I isotope [41]. The initial dilution for primary AS#438 was 1:2500. The minimum detection limit calculated for RIA-438 was 2.1 ng/mL. The intra- and inter-assay coefficients were 4.2% and 4.5%, respectively. Plasma PAG concentrations that were over 1 ng/mL were considered indicative of the presence of a secreting trophoblast. 

### 2.4. Isolation of PBMC and PMN

The blood samples (10 mL) were taken from the external jugular vein and placed in vacutainer tubes containing anticoagulant EDTA and processed within 1 h. PBMC were isolated by density gradient (Lymphoprep™; 1.077 g/mL; AXIS-SHIELD). In briefly, 8 mL of Lymphoprep was placed into a 50 mL Falcon^®^ tube and an equal volume of whole blood diluted 1:1 with Hank’s Buffered Salt Solution (HBSS without Ca^2+^ and Mg^2+^) was carefully layered on the gradient. The Falcon^®^ tube, containing two-step gradient of Lymphoprep and blood, was centrifuged at 800× g for 30 min and then washed twice with HBSS. The PMN and erythrocytes sedimented through the Lymphoprep layer during centrifugation. The erythrocytes were then lysed with 1:10 v/v of 1× Ammonium Chloride lysing solution (10× stock solution: NH_4_Cl 80 g, KHCO_3_ 10 g, Na_4_EDTA 3.7 g, final volume 1 L) and washed once with Phosphate Buffer Solution (PBS). The supernatant was aspirated carefully without disturbing the pellet and resuspended in PBS. The purity of the PBMC and PMN fractions were evaluated by flow cytometry. Cell subtypes were identified based on their FSC and SSC scatter, and auto-fluorescent properties. The purity of PBMC and PMN was 99% and 80%, respectively.

### 2.5. RNA Isolation Reverse Transcription and qPCR

Total RNA was isolated from PBMC and PMN cells with RNeasy^®^ Micro or Mini Kit purification system (Qiagen, Hombrechtikon, Switzerland) following manufacturer’s recommendations. The concentration of RNA was checked by analyzing the OD260/OD280 ratio. The RNA quality was then determined based on agarose gel electrophoresis and the RNA quantity was evaluated by Qubit Fluorometric Quantitation (Life Technologies, Carlsbad, CA, USA). RNA was stored at −80 °C until use.

Total RNA (15 ng) was reverse transcribed with iScript cDNA Synthesis Kit (BioRad, Hercules, CA, USA) according to the manufacturer’s protocol. 

QPCR were performed according to the method described by [42]. The optimized qPCR assays contained 10 μL TaqMan Gene Expression Master Mix (Applied Biosystems, Foster City, CA, USA), 1 μL TaqMan Gene Expression Assays (Applied Biosystems, Foster City, CA, USA) and water to achieve in a final volume of 20 μL. The probe sequences that were used are listed in Table 1. All reagents were mixed and distributed into a 96-well qPCR plate before adding 4 μL of diluted cDNA (1:10 with water). Each sample was run in triplicate and the results were averaged. In parallel, to assess the non-specific amplicon (primer-dimer formation) or genomic DNA contamination, no template control and no reverse transcriptase control were evaluated. PCR reactions were performed on iCycler iQ (Bio-Rad, Hercules, CA, USA) with an initial incubation at 95 °C for 10 min, followed by 45 cycles at 95 °C for 15 s and 60 °C for 60 s, during which time fluorescence data were collected. Prior to sequencing, PCR products were purified by QIAquick PCR Purification Kit (Qiagen, Hilden, Germany) according to the manufacturer’s instructions. Normalization of qPCR data was performed referring the selected targets ACTB by using the 2^−ΔCt^ method (ΔCt = Ct target mRNA − Ct ACTB). The stability of the candidate reference genes was evaluated through the BestKeeper algorithm [36].

### 2.6. Statistical Analysis

Diagnostic graphics were used for testing assumptions and outliers. Because non-normality of the data was detected, Log(x + 1) transformation were used for analysis and the back-transformed estimated marginal means were presented. The figures show raw data. The data were analyzed using the Linear Mixed model in which animals and days were included as subjects and repeated factors, respectively. Sidak adjustment was used for carrying out multiple comparisons. The model for PAG-2 concentrations determined by the RIA system evaluated the main effects of time (5 levels: 0, 14, 18, 28, and 40 Days post AI), outcome (2 levels: Pregnant and Non-Pregnant), and the interaction between outcome and time. To analyse the expression of PAG-2 in PMN and PBMC, the model evaluated the effects of time (5 levels: 0, 14, 18, 28, and 40 Days post AI), outcome (2 levels: Pregnant and Non-Pregnant), cell type (2 levels: PMN and PBMC), and their interactions. In order to test the ability of PAG-2 expression in PMN and PBMC to discriminate between pregnant and non-pregnant outcomes in early pregnancy, we performed the Receiver Operating Characteristic (ROC) analysis and we determined the optimal cut-off by using the Youden index [43]. Finally, we calculated the sensitivity and specificity at each day and for all the parameters. Statistical analyses were performed with SPSS Statistics version 23 (IBM, SPSS Inc., Chicago, IL, USA). Statistical significance occurred when *p* < 0.05.

## 3. Results

By ultrasound examination, of the 32 inseminated buffaloes used in the present study, 14 were confirmed to be pregnant and 18 were non-pregnant.

### 3.1. PAG-2 Concentrations Determined by RIA System in Pregnant and Non-Pregnant Buffaloes

**Figure 1:**
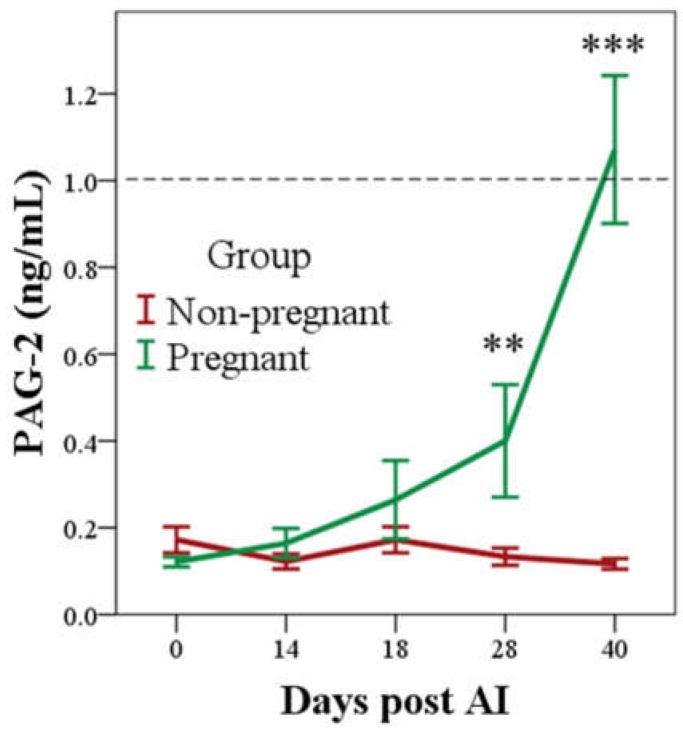
PAG-2 concentrations until Day 40 post AI in the pregnant and non-pregnant group (mean ± SE, row data) evaluated with the radioimmunoassay (RIA) system. *** *p* < 0.001. ** *p* < 0.01 Pregnant vs. Non-pregnant at each day (Sidak correction). The dotted line indicates the threshold for the pregnancy diagnosis (1.0 ng/mL).

No differences were observed in ln-PAG-2 between pregnant and non-pregnant groups until day 28 post AI, when it was 0.0 ± 0.0 ng/mL and 0.3 ± 0.1 ng/mL in non-pregnant and pregnant groups, respectively (*p* < 0.01; Figure 1). In non-pregnant buffaloes, ln-PAG-2 remained constantly close to 0.0 ng/mL throughout the observation period, while in pregnant animals they were significantly different to day 0, starting from day 28 post AI (*p* < 0.05). However, the mean concentrations reached the threshold for the diagnosis of pregnancy (1.0 ng/mL) only at day 40 post AI (Figure 1), when sensitivity and specificity were 78.6% and 100.0%, respectively (Table 2). 

### 3.2. *PAG2* Expression in PMN and PBMC in Pregnant and Non-Pregnant Buffaloes

The Basic Local Alignment Search Tool analysis of the amplicon sequence for the retrieval of the gene sequences showed 100% similarity with the sequences *PAG2* (ID: NM_176614.1) and *ACTB* (Bt03292796_gH).

**Figure 2:**
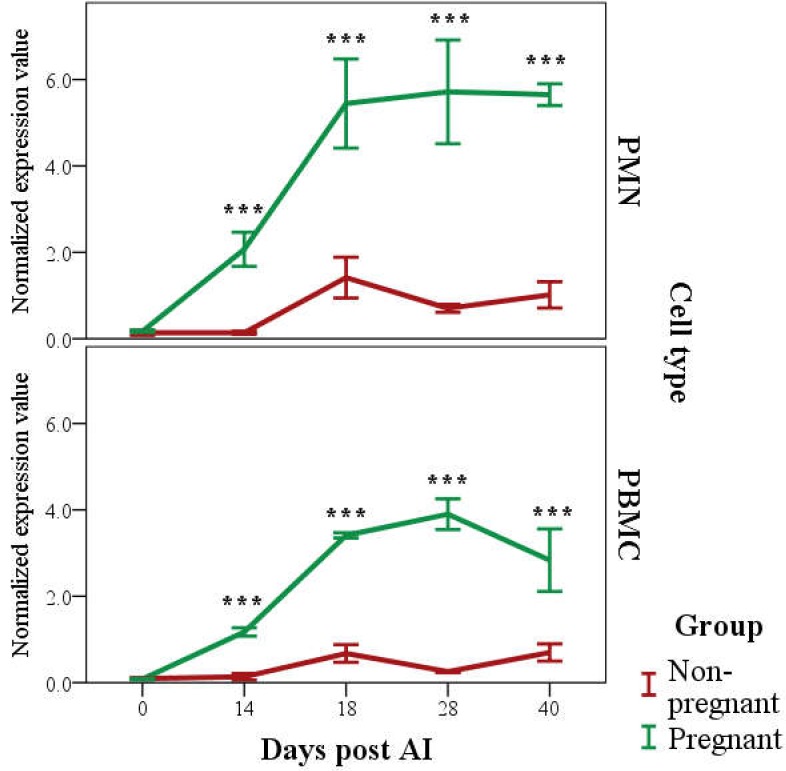
*PAG2* expression in granulocytes (PMN, 2^−ΔCt^; Upper Panel) and peripheral blood mononuclear cells (PBMC, 2^−ΔCt^; Lower Panel) until Day 40 post AI in the Pregnant and Non-pregnant group (mean ± SE, raw data). Differences, in both PMN and PBMC expression, between the Pregnant and Non-pregnant group as well as with respect to Day 0, starting from Day 14 post artificial insemination (AI; *** *p* < 0.001 Pregnant vs. Non-pregnant and each day of observation vs. Day 0).

Expression of *PAG2* (as 2^−ΔCt^) was affected by Time (*p* < 0.001), Group (*p* < 0.001), Cell type (*p* < 0.001), and interaction between Time and Group (*p* < 0.001; Figure 2). The estimated marginal means of expression of *PAG2* was higher in Pregnant than Non-pregnant group (2.3 ± 0.1 and 0.45 ± 0.1, respectively; *p* < 0.001) and in PMN than PBMC (1.5 ± 0.1 and 0.9 ± 0.1, respectively; *p* < 0.001). 

Differences both in PMN and PBMC expression of *PAG2* between the Pregnant and Non-pregnant groups was significant from Day 14 (*p* < 0.001; Figure 2). Similarly, the differences with respect to Day 0 started from Day 14 for both PMN and PBMC (*p* < 0.001; Figure 2).

**Figure 3:**
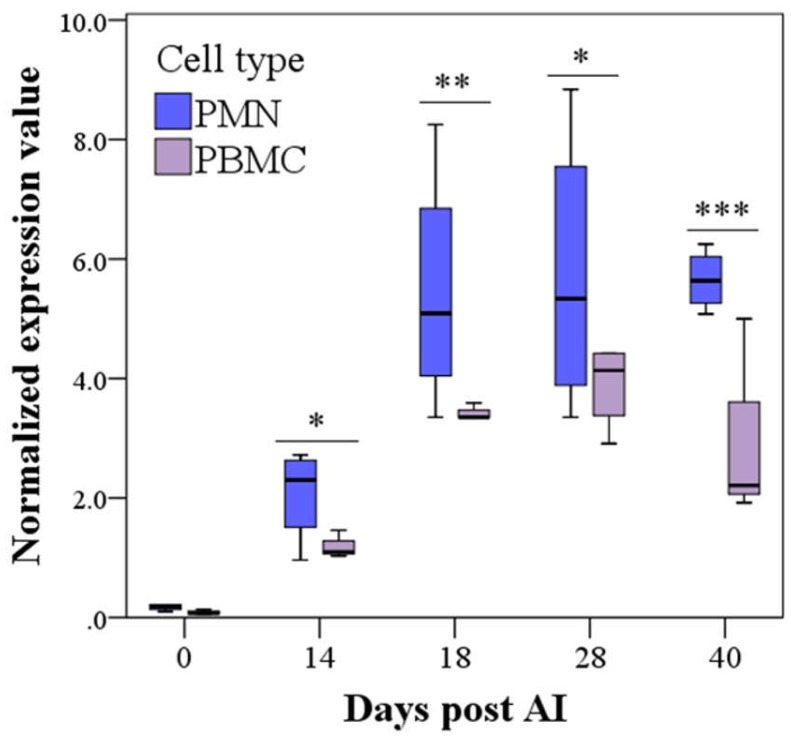
*PAG2* expression in granulocytes (PMN, 2^−ΔCt^) and peripheral blood mononuclear cell (PBMC, 2^−ΔCt^) until Day 40 post AI in Pregnant buffaloes (mean ± SE, raw data). *PAG2* expression was higher in PMN than PBMC from Day 14 post artificial insemination (AI; *** *p* < 0.001, ** *p* < 0.01, * *p* < 0.05 PMN vs. PBMC for each day).

Multiple comparisons also showed that in the Pregnant group, PMN was higher than PBMC starting from Day 14 (*p* < 0.05), although, PMN showed higher variability (Figure 3). 

Both PMN and PBMC discriminated perfectly between pregnant and non-pregnant buffaloes from Day 14 until the last day of observation (AUC = 1.000; CI 95%: 1.000–1.000; *p* < 0.05). The cut off was 0.59 and 0.70 at Day 14, 3.04 and 2.27 at Day 18, 2.10 and 1.62 at Day 28, and 3.50 and 1.60 at Day 40 post AI for PMN and PBMC, respectively (sensitivity = 100%, specificity = 100%).

## 4. Discussion

To our knowledge, no studies have been carried out on the mRNA expression of *PAG2* in peripheral blood leukocytes. The rationale for this work was to verify the expression of *PAG2* mRNA in maternal blood cells. We observed an early expression of *PAG2* mRNA, both in PMN and PBMC, at Day 14 post AI in all buffaloes that were diagnosed pregnant, although the expression was higher in the PMN than in the PBMC throughout the period of observation. This early expression confirmed our previous observations on this species [29]. We found that *PAG2* mRNA expression in maternal blood was detected at 18 Days after AI. These findings were in agreement with Xie et al. [3], who reported the expression of PAG–2 mRNA in bovine placenta as early as 17–19 days of pregnancy, Garbayo et al. [7] at 18 days in caprine and Green et al. [8] at 13 days of gestation in sheep.

The peri-implantation period is an extremely delicate time in the establishment of pregnancy in which various mechanisms are involved in maintaining a functional corpus luteum. It is generally accepted that pregnancy recognition in domestic ruminants is a local phenomenon involving IFN-stimulated genes (*ISG*). Recently, various works have demonstrated the presence of a viable conceptus by measuring levels of *ISG* in maternal peripheral blood leukocytes 15 days after insemination in sheep [44], 18 days after AI in dairy cattle [45] and between 15 and 20 days after AI in beef cattle [46]. The interferon tau (IFN-τ) is the major paracrine factor involved in pregnancy recognition in ruminant species [47,48,49]. Both PAG-2 and IFN-τ are Day 7/Day 14 and Day 14/Day 21 up-regulated genes in bovine conceptus, respectively [50]. Our study has highlighted the presence of mRNA *PAG2* expression in peripheral leukocytes, like *ISG*, therefore our hypothesis is that this glycoprotein could be involved in the process of maintaining the functional corpus luteum, and therefore, in the process of establishing the pregnancy. Moreover, different authors [51,52,53,54,55] have hypothesized that PAGs may also have an indirect role in the complex process of maternal recognition and maintenance of pregnancy through the increase in prostaglandin E_2_ and progesterone synthesis [56,57]. Furthermore, PAGs may have an additional role in inducing the increase in GPC-2 (granulocyte chemotactic protein-2) by bovine endometrial cells, which is involved in the establishment of pregnancy [58,59]. It remains to be clarified why the expression of *PAG2* mRNA appears to be higher in PMN than in PBMC.

To our knowledge, this is the first work in which the plasma concentration of PAG molecules during the gestation period was measured using antibodies raised against PAG-2 in buffalo, although, there are various works in which this method has been reported for bovine species [31,32,34,35]. Although significant differences were found starting from Day 28 post AI, our results show that the threshold value for pregnancy diagnosis (1.0 ng/mL) was only reached at Day 40 post AI, as previously reported for bovine species in [31,32,34,35]. Therefore, this work confirmed that in buffalo as well, the pre-eminent PAG radioimmunoassay method to diagnose pregnancy utilizes antibodies raised against PAG-1, as reported in our previous work [26,29,30].

## 5. Conclusions

In conclusion, the plasma PAG-2 threshold of 1.0 ng/mL was reached a Day 40 post AI in pregnant water buffalo, whereas expression of *PAG2* mRNA at Day 14 post AI was observed in peripheral blood leukocytes. Our results suggest that although further research is undoubtedly required to confirm these findings, *PAG2* mRNA in peripheral blood leukocytes could be used to better understand the role that PAGs play during pregnancy in buffalo, and even in other ruminant species.

Furthermore, in the future, the quantification of *PAG2* mRNA expression in blood leukocytes could be used to differentiate pregnant and non-pregnant buffaloes starting from the second week post AI, minimizing the time for resynchronization and rebreeding.

## Figures and Tables

**Table 1:** TaqMan Gene Expression assays used in the studies are listed by assay numbers. The TaqMan Gene Expression assay is composed of a pair of unlabelled PCR primers and a probe with a FAM dye label on the 5′ end, and a minor groove binder (MGB) non-fluorescent quencher (NFQ) on the 3′ end.

Name	Sequence	Exons Connected	Amplicon
Pregnancy-Associated Glycoprotein 2 (*PAG2*)	BOS	9-9	127 bp
TaqMan Bt03292796_gH	NM_176614.1		
Actin beta(*ACTB*)	BOS	4-5	144 bp
TaqMan Bt03279175_g1	NM_173979.3		

**Table 2:** Sensitivity, specificity, and accuracy of PAG-2 determined by RIA for pregnancy diagnosis in buffaloes 14, 18, 28, and 40 days post-insemination (threshold = 1.0 ng/mL).

Day	Diagnosis	Outcome ^1^		Sensitivity	Specificity	Accuracy
		Non-Pregnant (*n* = 18)	Pregnant (*n* = 14)			
14	Non-Pregnant	18 (100.0%)	14 (100.0%)	0.00%	100.00%	56.30%
	Pregnant	0 (0.0%)	0 (0.0%)			
18	Non-Pregnant	18 (100.0%)	13 (92.9%)	7.10%	100.00%	59.40%
	Pregnant	0 (0.0%)	1 (7.1%)			
28	Non-Pregnant	18 (100.0%)	11 (78.6%)	21.40%	100.00%	65.60%
	Pregnant	0 (0.0%)	3 (21.4%)			
40	Non-Pregnant	18 (100.0%)	3 (21.4%)	78.60%	100.00%	90.60%
	Pregnant	0 (0.0%)	11 (78.6%)			

^1^ Determined ex post on the basis of pregnancy diagnosis by ultrasonography at day 28 and 40.
